# Supplementary material for: Drying-Time Study in Graphene Oxide
Source: Nanomaterials (Basel). 2021 Apr 19;11(4):1035. doi: 10.3390/nano11041035 (PMC8072584; doi:10.3390/nano11041035)
Supplement: Supplementary file 1 [file nanomaterials-11-01035-s001.zip › nanomaterials-1178446-supplementary.pdf]

Drying-time study in graphene oxide

Talia Tene, Marco Guevara, Andrea Valarezo, Orlando Salguero, Fabian Arias Arias, Melvin Arias, Andrea Scarcello, Lorenzo S. Caputi and Cristian Vacacela Gomez

Supplementary Figures

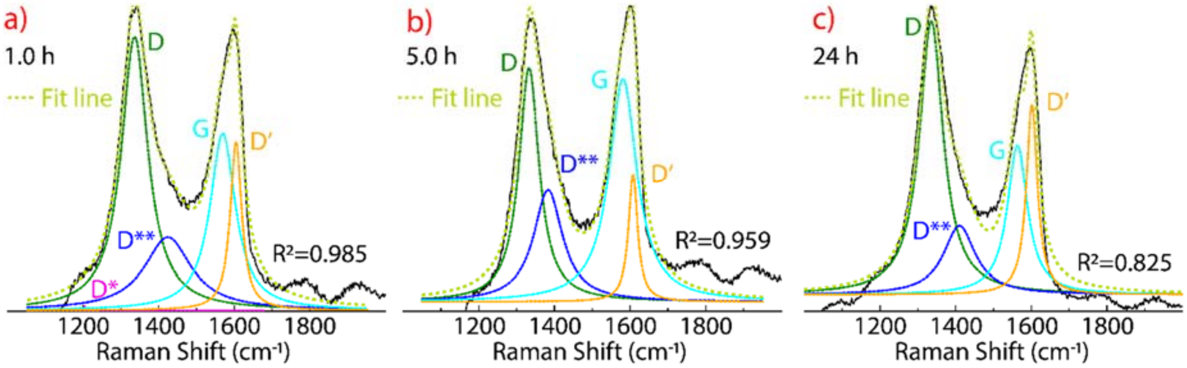

Figure S1. Raman spectra of rehydrated GO samples (1 h, 5 h, 24 h) from 1000 to 2000 cm^{-1} recorded using 532 excitation laser. The intensity was normalized by the most intense peak and the fitting of the peaks using Lorentzian functions.

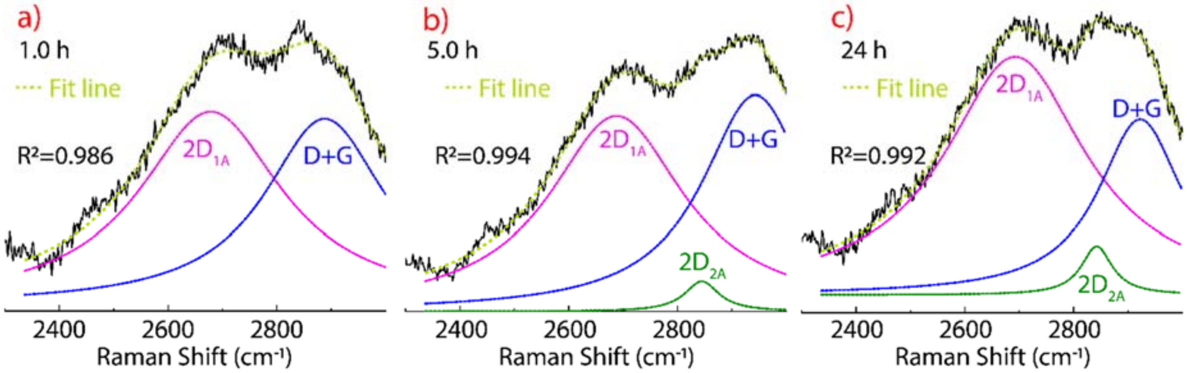

Figure S2. Raman spectra of rehydrated GO samples (1 h, 5 h, 24 h) from 2300 to 3000 cm^{-1} recorded using 532 excitation laser. The intensity was normalized by the most intense peak and the fitting of the peaks using Lorentzian functions.

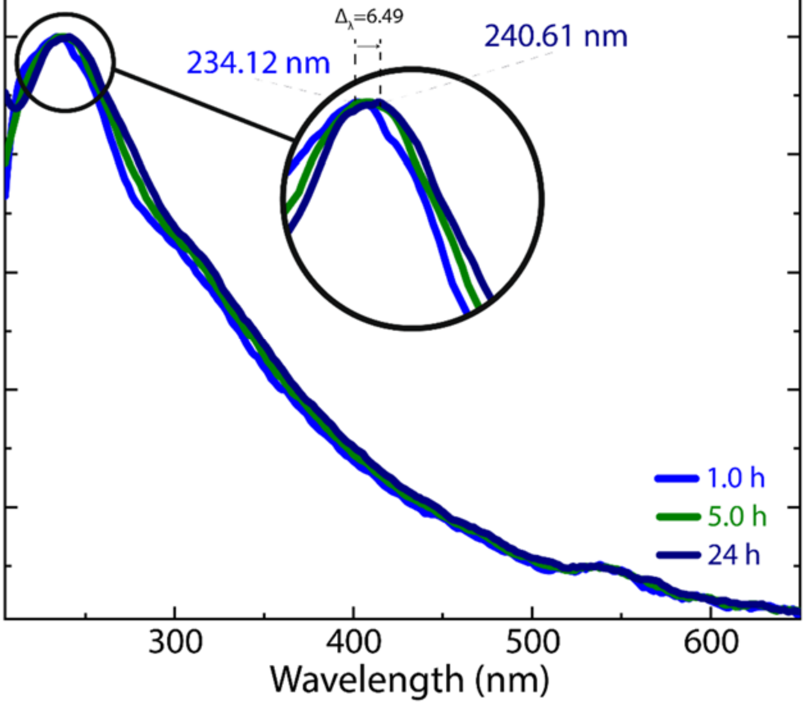

Figure S3. UV-vis spectra of rehydrated GO samples (1 h, 5 h, 24 h).
